# TERT Promoter Mutation in Benign and Malignant Salivary Gland Tumors; A Cross-Sectional Study

**DOI:** 10.30699/IJP.ijp.2023.556651.2927

**Published:** 2023-03-23

**Authors:** Ali Zare-Mirzaie, Shamim Mollazadehghomi, Seyed Mohammad Heshmati, Amirhosein Mehrtash, Shabnam Mollazadehghomi

**Affiliations:** 1 *Department of Pathology, School of Medicine, Iran University of Medical Sciences, Tehran, Iran*; 2 *Cellular and Molecular Research Center, Research Institute for Non-Communicable Diseases, Qazvin University of Medical Sciences, Qazvin, Iran*; 3 *Molecular Medicine Department, Biotechnology Research Center, Pasteur Institute of Iran, Tehran, Iran*

**Keywords:** DNA sequencing, Salivary gland tumors, TERT promoter mutation

## Abstract

**Background & Objective:**

Telomere-related tumorigenesis mechanisms in the salivary gland, including mutation in the promoter region of TERT, have been rarely investigated. Therefore, the present study aimed to investigate the mutation in the promoter region of TERT in benign and malignant salivary gland tumors.

**Methods:**

This was a descriptive-analytical cross-sectional study. Tissue samples of 54 patients with primary salivary gland tumors sent to the pathology department of Rasool-e-Akram Hospital from September 2017 to September 2021 were examined. Fifteen samples including two groups of the most common benign tumors (n=5; 3 pleomorphic adenomas and 2 Warthin tumors) and four groups of the most common malignant tumors (n=10; 3 mucoepidermoid carcinomas, 3 adenoid cystic carcinomas, 2 acinic cell carcinoma, and 2 salivary duct carcinoma) were selected. The promoter region of TERT, including well-known hot spot regions, is sequenced using the Sanger sequencing method. Data were analyzed using statistical software R version 4.1.2.

**Results:**

Of 15 salivary gland tumor specimens, consisting of 5 benign tumors and 10 malignant tumors after DNA sequencing, TERT promoter region mutation was only seen in one of the adenoid cystic carcinoma samples, located at -146 bp upstream from ATG (chr5: 1,295,250 C>T).

**Conclusion:**

TERT promoter mutation was not different in malignant and benign salivary tumors. Nonetheless, there are a few studies that report TERT promoter mutation in adenoid cystic carcinoma of the salivary gland, necessitating the need for further investigations.

## Introduction

The three main pairs of salivary glands (i.e., parotid, submandibular, and sublingual) (1) and the secondary salivary glands are located in the upper airway and gastrointestinal tract (2), especially in the oral cavity and oropharynx. A wide range of neoplasms with different clinical, histological and biological features can arise from the above-mentioned glands (3). These lesions are often a diagnostic challenge for pathologists due to inconclusive morphological findings (4). 

Salivary gland tumors are not very common and constitute 3 to 6% of head and neck neoplasms. The average annual incidence of benign tumors is 4.7 per 100,000 and 0.9 per 100,000 for malignant ones (5). Salivary gland tumors are more common in adults than children (6). The most common benign salivary gland tumor is pleomorphic adenoma (7). Besides, mucoepidermoid carcinoma (5) and adenoid cystic carcinoma (8) have been reported as the most common malignant tumors despite inconsistent reports. Mortality of salivary gland tumors varies depending on the stage and type of tumor lesions, as the average five-year survival of patients is reported to be about 72% (5). Furthermore, about 80% of suspected malignancies on biopsy are benign, and in some cases, benign specimens such as pleomorphic adenomas are included in the differential diagnosis of adenoid cystic carcinoma (9). Therefore, diagnosis, prognosis, and optimal treatment of salivary gland tumors have always been a challenge due to differences in subtypes and their behavior (10). A definitive diagnosis is based on histopathological findings, but may not help in all cases.

 In the fourth edition of the World Health Organization (WHO) regarding head and neck tumors, 31 unique epithelial tumors were identified, which include 20 malignant and 11 benign types (11). Immunohistochemistry and molecular tools might help for more accurate diagnosis, especially when faced with morphological overlap (12). Telomeres are well-known structures of the TTAGGG nucleotide sequence, which were first identified by Muller* et al.* in 1938 (13). The TERT promoter is considered the most important component that guides and regulates telomere length as well as telomerase activity, and therefore any alternation or mutation in this promoter would cause abnormality in telomere length and telomerase function (14). Two known mutations in the TERT gene have been identified: mutations (C228T) C> T and (C250T) C> T (15). The occurrence of these two mutations is the result of the replacement of thymine by organic cytosine in loci 124 and 146, respectively (16). Today, various studies have been performed to evaluate mutations in the TERT promoter region and their association with different cancers, such as melanoma and gastric, thyroid, or urothelial cancer, but very few studies have been performed regarding the association between these promoter mutations and salivary gland tumors. Therefore, the present study was performed to evaluate TERT Promoter mutations in benign and malignant salivary gland tumors.

## Material and Methods

This was a descriptive-analytical cross-sectional study. Tissue samples of 54 patients with primary salivary gland tumors (not metastatic/recurrent) sent to the pathology department of Rasool-e-Akram Hospital from September 2017 to September 2021 were examined. 

**Patients**

In the beginning, a checklist including the type of tumor (benign or malignant), information on age, sex, and patient history was filled. According to the inclusion and exclusion criteria, 54 samples were included; however, due to limited financial resources, 15 samples, two groups of the most common benign tumors (n=5; 3 pleomorphic adenoma and 2 warthin tumors) and four groups of the most common malignant tumors (n=10; 3 mucoepidermoid carcinoma, 3 adenoid cystic carcinoma, 2 acinic cell carcinoma, and 2 salivary duct carcinoma) were selected. After DNA extraction from paraffin blocks and polymerase chain reaction (PCR), the resulting products were subjected to genetic sequencing by the Sanger method, and genomic sequences (mutant and non-mutant) were recorded.

Inclusion criteria were as follows; patients without previous history of head and neck radiotherapy, those without malignancies other than salivary gland tumors, paraffin slices and blocks containing more than 80% of tumor tissue, paraffin slices and blocks containing a specific type of malignant or benign salivary gland tumor. 

Exclusion criteria were as follow; patients with a previous history of head and neck radiotherapy, those with malignancies other than salivary gland tumors, paraffin slices and blocks containing more than 20% of non-tumoral or necrotic tissue, paraffin slices and blocks containing a non-specific type of malignant or benign salivary gland tumor.

**DNA Extraction**

D.N.A. extraction from paraffin tissues was performed using the E.Z.N.A universal pathogen D.N.A. extraction kit (Omega Bio-Tek U.S.A.), as follows: 

First, six 5-micron slices were placed in a 1.5 mL microtube. One mL of xylene solution (Dr. Mojallali industrial chemical complex, IRAN.) was added to the microtube containing paraffin tissue. After 10 minutes at room temperature, the microtube containing paraffin tissue was centrifuged at 11,000 rpm and the supernatant was spilled. Then, one mL of 100% Merck alcohol was added to the microtube. At that time, the microtube was centrifuged at 11,000 rpm for 1 minute, and the supernatant was spilled. One mL of 100% alcohol (Merck, Germany) was added to the microtube again. The microtube was then centrifuged at 11,000 rpm for one minute, and the supernatant was spilled. The microtubes were incubated at 37°C for 15 minutes to dry the alcohol. Thereafter, 200 μL of TL buffer solution and 25 μL of proteinase K were added to the microtube and incubated at 55°C for 2 hours. Next, the microtubes were incubated at 90°C for 30 minutes. 220 μL of BL buffer solution was added to the microtube and incubated for 15 minutes at 70°C. Then, 220 μL of 100% alcohol was added to the microtube, and the contents containing the microtube were added to the HiBind D.N.A. column, centrifuged at 11,000 rpm for 1 minute, and the following solution was poured out. 500 μL of HBC wash buffer solution was added to the HiBind D.N.A. column, then centrifuged at 11,000 rpm for 1 minute, and the following solution was poured out. Afterward, 700 μL of DNA wash buffer solution was added to the HiBind D.N.A. column, then centrifuged at 11,000 rpm for 1 minute, and the following solution was poured out. Next, 700 μL of DNA wash buffer solution was added again to the HiBind D.N.A. column, then centrifuged at 11,000 rpm for 3 minutes and the following solution was poured out. Later, the HiBind D.N.A. column was transferred to a clean 1.5 mL microtube and 50 μL of elution buffer was added and then centrifuged at 11,000 rpm for 1 minute. Subsequently, the extracted DNA was read by a Nano-Drop (Thermo-Scientific, U.S.A.) to measure the concentration and purity and stored in a freezer at minus 20 degrees.

**PCR AND Sequencing**

TERT gene PCR was performed using the following materials for each sample:

1. HotStarTaq Master Mix/ Solis BioDyne 5X company/ 6 μL

2. Primer Forward and Reverse 10 pmol/microliter/ 1 μL

Forward primer sequence: CGGGCTCCCAGTGGATTCG

Reverse primer sequence: CAGCACCTCGCGGTAGTGG

**Table 1:** PCR temperature program

Final Stage	Cycling Stage 40 cycle			Initial denaturation stage
**72**	72	62	95	95
**10 min**	40 Sec	30 Sec	40 Sec	10 min

**Fig. 1:**
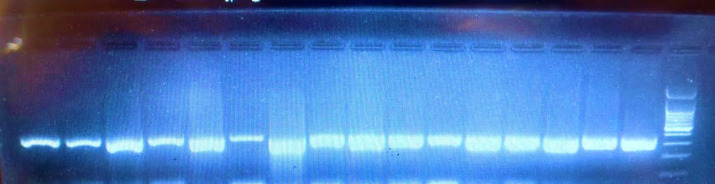
Electrophoresis gel band. Each band represents a specific DNA molecule that has a specific molecular weight. The right band of the image indicates the ladder and the second line is a positive control

Standard PCR temperature programming (Table 1) was as the following for the samples:

After examining PCR products on 1% gel electrophoresis (Figure 1), the PCR product, along with Forward and Reverse primers, were subjected to the Sanger sequencing by 3130 XL Applied bioSyestems-ABI (USA). Then, sequencing data were interpreted based on Forward and Reverse primers in Chromas software version 2.6.6.

**Data Analysis**

Data were analyzed using statistical software R version 4.1.2. The analysis results were reported as frequency percentage for qualitative and mean and standard deviation for quantitative variables. Chi-Square and Fisher statistical tests were used to evaluate the association between qualitative variables and the t-test for quantitative ones. The significance level was considered as P-value <0.05**.**

## Results

Fifteen (nine men and six female) patients with a mean ± SD age of 45.4 ± 3.1 years were included. The youngest patient with a mucoepidermoid carcinoma was a 19-year-old woman, and the oldest one with an adenoid cystic carcinoma was an 86-year-old man. Data on age, sex, and tumor location, microscopic view of the tumor, and the results of genomic sequencing were presented in Tables 2, 3, 4, and 5.

**Table 2:** Clinical information and results of TERT promoter genomic sequencing in pleomorphic adenomas tumors

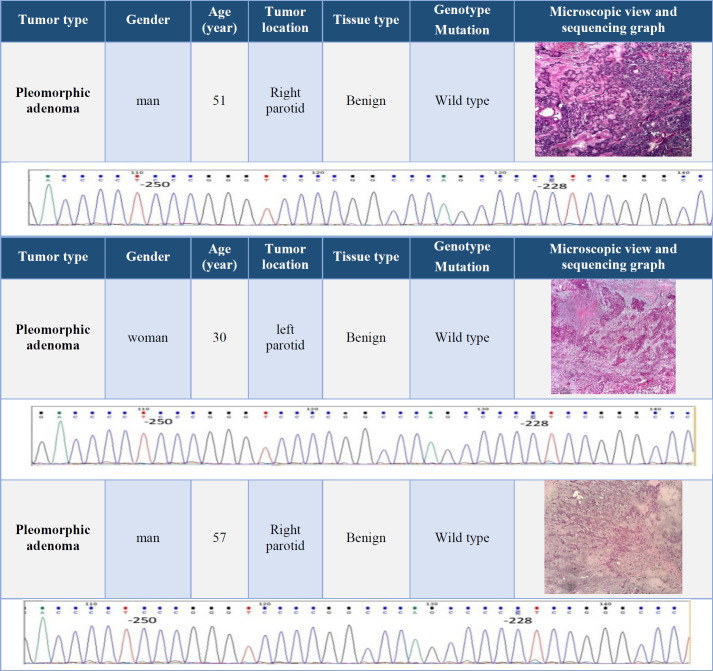

**Table 3:** Clinical information and results of TERT promoter genomic sequencing in Warthin tumors

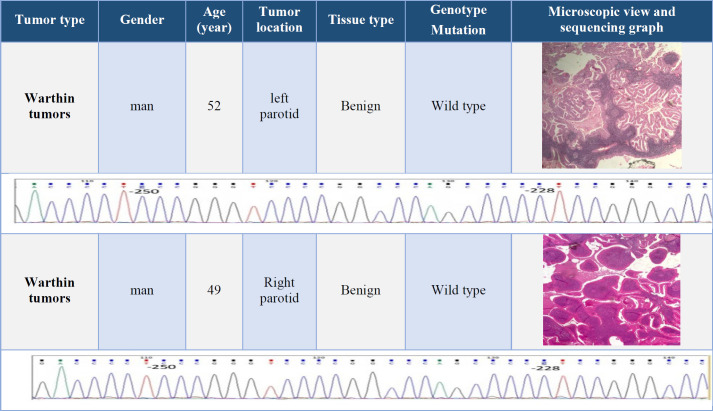

**Table 4:** Clinical information and results of TERT promoter genomic sequencing in Mucoepidermoid carcinoma and Adenoid cystic carcinoma

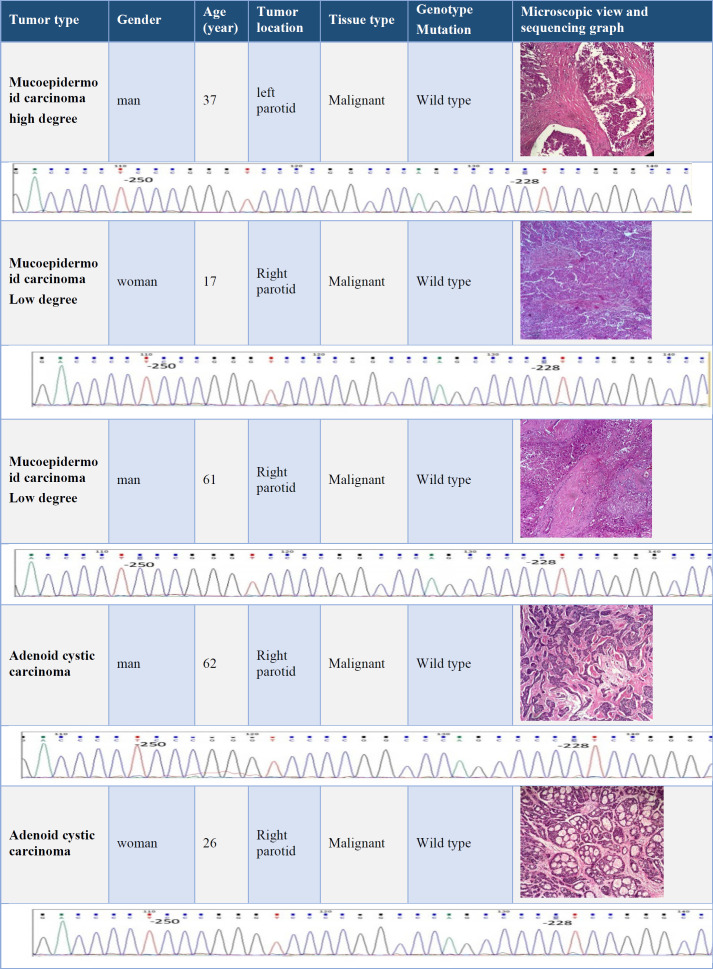

**Table 5:** Clinical information and results of TERT promoter genomic sequencing in Acinic cell carcinoma and ductal carcinoma

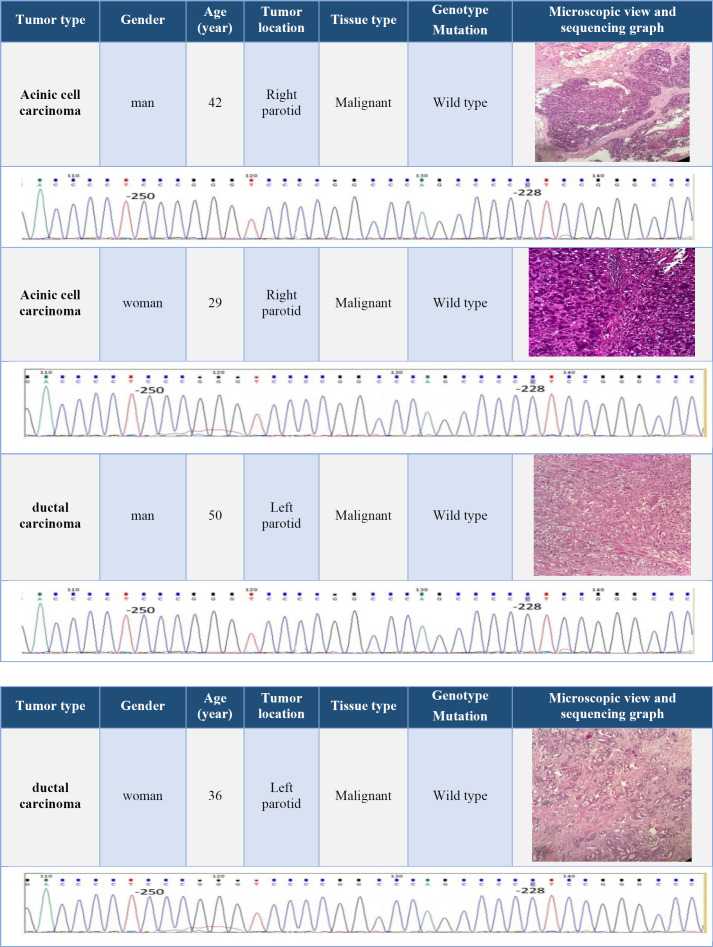

Finally, a mutation at 146 nucleotide position (C250T) was detected in a sample of primary adenoid cystic carcinoma of parotid carcinoma in an 82-year-old man. However, There was no statistically significant difference between benign and malignant tumors (Table 6) (*P*>0.05).

**Table 6:** Comparison of genotypes (wild/mutant) of TERT promoter in benign and malignant salivary gland tumors

Tumor type	Sample (%)	mutants T.E.R.T. promoter (%)	Wild T.E.R.T. promoter (%)	Fisher's exact test
Benign	5 (33.33)	0	5 (100)	1.00
Malignant	10 (66.66)	1 (10)	9 (90%)	
Total	15 (100)	1 (6.66)	14 (93.33)	

## Discussion

To the best of our knowledge, this is one of the rare investigations to report TERT promoter mutation in adenoid cystic carcinoma of the salivary gland (50, 51), necessitating the need for further investigations. Over the past two decades, various studies have been conducted on the epidemiology of salivary gland tumors. According to the present findings, most salivary gland tumors were benign (70%), which was similar to studies in Iran, Cameroon, Brazil, and the United Kingdom, in which the rate of malignant tumors ranged from 51% to 84% of whole salivary gland tumors (17-20). However, Taghavi* et al.* reported that malignant tumors of the salivary glands had a higher percentage than benign tumors, which may be due to the higher percentage of submucosal salivary gland tumors in this study (75% of gland samples) (21). According to our study on 54 samples of primary salivary gland tumors (not metastatic/recurrent), the most common benign and malignant tumors of the salivary glands were pleomorphic adenoma (17, 21-23) and mucoepidermoid carcinoma (17, 21, 24, 25), which is consistent with the results of other investigations. 

On the other hand, in some previous studies, adenoid cystic carcinoma (26-28) was the most common malignant tumor of the salivary glands. In general, in previous studies, mucoepidermoid carcinoma, adenoid cystic carcinoma, acinic cell carcinoma, and adenocarcinoma were the most common malignant tumors of the salivary glands. During the last 15 years, extensive studies have been conducted on the role of TERT promoter gene mutation in increasing telomere length and its effect on cancer. However, the role of these mutations has been examined in many tumors of the central nervous system (29-31), thyroid (32, 33), urinary tract (34, 35), cutaneous melanoma (36, 37), sarcoma (38, 39), ovary (40) and gastrointestinal tumors (41, 42). 

Adenoid cystic carcinoma is a rare and often resistant tumor with a high rate of metastatic spread. Adenoid cystic carcinoma mainly occurs in the salivary glands but may originate from the trachea, lungs, breast, etc. (8) 

More than 50% of adenoid cystic carcinoma recur over time or develop distant metastases, which are generally incurable. Therefore, the overall prognosis of this tumor remains poor, and its overall survival rate is 25-40% (43). KIT, a membrane tyrosine kinase receptor, is overexpressed in adenoid cystic carcinoma and can be detected by immunohistochemistry. Imatinib (a tyrosine kinase inhibitor) was used in some patients with non-removable metastatic adenoid cystic carcinoma who overexpressed CD117. However, the response to treatment has been disappointing (44). Today, various therapeutic approaches are being developed to target tumors with TERT mutations, including 1. Immunotherapy using TERT as a Tumor‐Associated Antigen (45), 2. Antisense oligonucleotide-based therapies (46) and 3. TERT promoter‐directed cytotoxic molecules (47).

In Kim* et al.* study (48), 36 patients with salivary gland tumors, including 31 benign tumors and 5 malignant tumors, were examined for TERT promoter mutation and telomere length. In Kim's study, telomere length in malignant tumors was evaluated. They were shorter, although there was no significant difference between benign and malignant groups, and no TERT promoter gene mutation was observed in any of the samples. In the present study, compared to the study of Kim *et al.*, a doubled number of malignant samples (10 samples compared to 5 samples) were included, and also in our study, there were 2 samples of mucoepidermoid carcinoma (as the most common malignant tumor of the salivary glands in most studies) which was absent in Kim study. In our study, telomere length was not measured. However, unlike the study by Kim* et al.*, a TERT promoter mutation in a malignant sample of cystic adenoid carcinoma was identified. 

In the case report of Cormier* et al.*, (49), a 76-year-old woman with a left parotid mass with a primary diagnosis of cellular pleomorphic adenoma recurred as left parotid mass and cervical lymphadenopathy after 9 months of surgery and, a myoepithelial carcinoma (MECA ex-PA) was detected using pathological blocks on revisions. In the genomic sequencing of this patient's blocks, a mutation at the 124 nucleotide site (C228T) in the TERT promoter gene was detected, which is a very rare finding. This indicates the importance of doing more research on TERT promoter gene mutations in patients with salivary gland tumors.

The present study had some limitations. Some of the most important limitations of this study were the number of patients due to the low prevalence of salivary gland tumors and limited financial resources and laboratory equipment. It is suggested to perform similar studies on malignant salivary gland tumors with a higher statistical population and multicentric collaboration. 

## Conclusion

A mutation at the 146 nucleotide site (C250T) in the TERT promoter gene was detected by the Sanger sequencing method in an 82-year-old man with primary adenoid cystic carcinomas. Although in our study the incidence of TERT promoter gene mutation in two groups of malignant and benign salivary gland tumors was not statistically different (*P*>0.05), This study can serve as a guide in the field of genetic-molecular studies on malignant salivary gland tumors with a higher statistical population. In order to more accurately diagnose salivary gland tumor specimens.

## Conflict of Interest

The Authors declare that there is no conflict of interest.

## Author's Contribution

AZ designed the study plan, SM collected the data and analyzed the data and drafted the paper. All authors have accepted responsibility for the entire content of this manuscript and approved its submission.

## Financial Support and Sponsorship:

The study was supported financially by the Iran University of Medical Sciences.
